# Non-VKA Oral Anticoagulants: Accurate Measurement of Plasma Drug Concentrations

**DOI:** 10.1155/2015/345138

**Published:** 2015-05-19

**Authors:** Jonathan Douxfils, Helen Mani, Valentine Minet, Bérangère Devalet, Bernard Chatelain, Jean-Michel Dogné, François Mullier

**Affiliations:** ^1^Department of Pharmacy, University of Namur, Namur Thrombosis and Haemostasis Center (NTHC), Namur Research Institute of Life Sciences (NARILIS), 5000 Namur, Belgium; ^2^Department of Haemostasis, University Hospital Frankfurt, 60596 Frankfurt am Main, Germany; ^3^Department of Haematology, CHU Dinant-Godinne UCL Namur, Namur Thrombosis and Haemostasis Center (NTHC), Namur Research Institute of Life Sciences (NARILIS), 5530 Yvoir, Belgium; ^4^Haematology Laboratory, CHU Dinant-Godinne UCL Namur, Namur Thrombosis and Haemostasis Center (NTHC), Namur Research Institute of Life Sciences (NARILIS), 5530 Yvoir, Belgium

## Abstract

Non-VKA oral anticoagulants (NOACs) have now widely reached the lucrative market of anticoagulation. While the marketing authorization holders claimed that no routine monitoring is required and that these compounds can be given at fixed doses, several evidences arisen from the literature tend to demonstrate the opposite. New data suggests that an assessment of the response at the individual level could improve the benefit-risk ratio of at least dabigatran. Information regarding the association of rivaroxaban and apixaban exposure and the bleeding risk is available in the drug approval package on the FDA website. These reviews suggest that accumulation of these compounds increases the risk of experiencing a bleeding complication. Therefore, in certain patient populations such as patients with acute or chronic renal impairment or with multiple drug interactions, measurement of drug exposure may be useful to ensure an optimal treatment response. More specific circumstances such as patients experiencing a haemorrhagic or thromboembolic event during the treatment duration, patients who require urgent surgery or an invasive procedure, or patient with a suspected overdose could benefit from such a measurement. This paper aims at providing guidance on how to best estimate the intensity of anticoagulation using laboratory assays in daily practice.

## 1. Introduction

The fact that non-VKA oral anticoagulants (NOACs) do not require frequent biological monitoring led the products' manufacturers to put forward this advantage as one of their main selling points. However, the “one dose fits to all” policy suffers from several criticisms both on the scientific literature side [1, 2] and on the regulatory side. Effectively, dose adaptations are proposed by the different regulatory agencies for dabigatran etexilate, rivaroxaban, and apixaban regarding the degree of renal insufficiency [3–8]. Moreover, even if they are less frequent compared with vitamin K antagonists (VKA), several drug-drug interactions have been listed [9], such as concomitant treatment with drug affecting the glycoprotein-P (P-gp) and/or CYP3A4/5. These interactions also led to dose adaptation or to a nonrecommendation of concomitant administration [3–8]. In addition, several conditions such as renal and hepatic function, age, gender, and body weight impact on the exposure [10–12]. It is acknowledged that some of these factors do not or minimally alter pharmacokinetics of these agents if taken independently. However, taken concomitantly, they may have clinical implications if drugs are accumulating and/or are less easily eliminated or metabolized.

Different authors have already pointed out the fact that one should not abolish the opportunity to further improve the benefit-risk balance of NOACs. This could require the use of occasional monitoring to assess the response at the individual level to ensure that the optimal dose is delivered, taking into account the patient characteristics [1, 2, 13, 14]. Accumulative data of case reports from patients with major bleeding and regulatory documents associated supratherapeutic level of anticoagulants and bleeding outcomes [15–18]. This suggests that certain populations, especially those with impaired renal function and the elderly, may accumulate the drug to such extent that they put them at an increasing risk of bleeding event [19–23].

Thus, while the absence of monitoring was one of the main selling arguments in favour of NOACs, it appears that occasional monitoring could be implemented in routine practice to ensure a safe and efficient treatment. However, although the gold standard to measure plasma drug concentrations is mass-spectrometry (LC-MS/MS), several limitations, that are, the availability, the laboratory experience, the local validation, and the turn-around time in emergency situations, restrict its implementation in the clinical setting. Hence, less restrictive coagulation tests have been tested to assess the pharmacodynamics of NOACs.

This paper aims at providing an overview of our current knowledge regarding how to accurately estimate plasma drug concentrations or the intensity of anticoagulation using conventional or more specific coagulation assays.

## 2. Rationale for Dose Tailoring NOACs in Specific Situations

This question is subject to a debate in the literature. Some authors argue that the anticoagulant effect of a fixed dose of all of these new agents is highly predictable and claim that there is no evidence that (re-)thrombosis or bleeding correlates with any measured biologic activity or drug concentrations in plasma [24]. In addition, there is not yet evidence that an individualized regimen with occasional monitoring and dose tailoring is safer or more effective than a standard dose regimen. Others argue that, in “real life,” opposing to the clinical trial setting, there are abundant factors, such as body weight, age, renal and liver function, concomitant interacting drug and genetic variants, and probably many other unknown causes that influence the plasma levels and the anticoagulant effect of all of these agents. The most conservative approach would suggest that fine tunings of the dosage to the individual needs might be preferable [13].

Since these claims, information has been released in the literature regarding dabigatran etexilate, the first NOAC that reached the market and therefore the most studied to date. Thus, recent analyses of the RE-LY trial, evaluating dabigatran etexilate versus warfarin in patients with nonvalvular atrial fibrillation, revealed a high interindividual variability. Genetic variants were investigated to explain a part of the large interindividual variability and it was demonstrated that the rs2244613 intronic SNP to the esterase gene* CES1* was associated with a decreased trough concentrations and a decreased risk of bleeding [15]. In addition, a reanalysis of the RE-LY trial mentions that compared with the median *C*_trough_ concentration of 88 ng/mL, the rate of major bleeding doubled at a concentration of 210 ng/mL [16]. A higher residual *C*_trough_ plasma concentration also reduced the risk of stroke but to a lesser extent compared with the increase in major bleeding. The authors concluded that, in patients with the highest risk for events, such as the very elderly and/or those with poor renal function, an adjustment of dabigatran dose to optimize exposure might improve the benefit-risk balance.

For rivaroxaban and apixaban, data on drug levels associated with therapeutic or harmful ranges are currently lacking in the literature, but information on the risk of bleeding and recurrence of thrombosis associated with plasma drug levels can be found in the FDA-Clinical Pharmacology and Biopharmaceutics Review(s).

For patients treated with rivaroxaban 20 mg* od* for the treatment of acute DVT, the 90th percentile of rivaroxaban plasma concentrations measured at trough (24 hours after the previous dose) was about 249 ng/mL [12]. Similar concentrations were found in the setting of NVAF. The FDA-Clinical Pharmacology Biopharmaceutics Review(s) of Xarelto also mentions that a two-fold increase in exposure due to intrinsic and extrinsic factors will increase the risk of major bleeding by 50% [17]. For apixaban, no data regarding the plasma trough or max level versus bleeding or recurrence of thrombosis are available. However, it is mentioned that the risk of major bleeding increased with the exposure to apixaban, measured as AUC_SS_ [10, 18].

Of course, the overall performance of NOACs in the large phase-III clinical trials is noninferior or better, compared to INR-adjusted warfarin. However, we should keep in mind that these were carefully selected patients to begin with while bleeding and other side effects were still encountered at significant percentages [25].

## 3. In Which Patients and When Should We Measure Plasma Concentrations or Estimate the Intensity of Anticoagulation?

There are several situations in which it may be of assistance to assess the anticoagulant effects of NOAC [26]. In addition to the above-mentioned reasons, biological monitoring would also be valuable in acute situations such as the following [2, 14, 16, 27–29]: recurrence of thrombosis or bleeding, before surgery or invasive procedure, before fibrinolytic therapy of acute ischaemic stroke, in case of bridging therapy, or also in case of cardioversion.

Moreover, it could be useful in patients with risk factors that could lead to an accumulation or to insufficient levels of NOACs such as in patients with renal or hepatic impairment, in polymedicated patients with multiple drug-drug interactions, in patients with genetic variants interfering on the metabolic pathways (e.g., rs2244613 intronic SNP to the esterase gene* CES1* for dabigatran: no mutations linked to changes in the pharmacokinetics are known for the other NOACs at that time), or in patients with extreme body weight. These situations are summarized in Table 1.

The collection of information is mandatory when prescribing a biological test for patients treated with NOACs. Therefore, information on the age, the weight and the renal function of the patient, the concomitant therapies, the dosage of the NOAC, the number of administration(s) per day, the delay since the last administration, and the indication should be collected in order to obtain sufficient materials to appropriately interpret the results of such tests.

## 4. How to Accurately Estimate Plasma Drug Concentrations?

### 4.1. Sample Acquisition

Sample acquisition is of a great importance since it was proven that each component of the specimen collection system (needle gauge, composition of the collecting tube, and concentration of sodium citrate) might potentially impact the results for coagulation testing. Blood should be taken with 21-gauge needles at the antecubital veins to avoid activation of the coagulation due to a slower rate of blood flow (with >25-gauge needles) or haemolysis of the sample due to turbulence of flow through the needle (with <16-gauge needle).

Samples for plasma-based haemostasis testing should be anticoagulated with sodium citrate. The World Health Organization (WHO) and Clinical Laboratory Standards Institute (CLSI) recommend 105 to 109 mmol/L (3.13% to 3.2%) of the dehydrate form of trisodium citrate, buffered or nonbuffered as the anticoagulant of choice for haemostasis testing. The blood anticoagulant ratio is understandably a source of variability and collection containers that are underfilled contain proportionally more sodium citrate per volume of plasma leading to longer clotting times. As for routine coagulation testing, cold storage of citrate whole blood should be avoided since it may lead to platelet activation, activation of FVII, and significant time-dependent loss of both FVII and VWF [30].

### 4.2. Specimen Processing

The centrifugation should take place as soon as possible after the blood sampling during no less than 15 minutes at 1500 ×g. Double centrifugation can be performed to ensure the plasma is platelet-poor. The use of relative centrifugal forces (RCFs) greater than 1500 ×g is not recommended as this may induce platelet activation and red blood cell lysis.

### 4.3. Stability and Storage of Plasma Samples

There is currently no recommendation based on samples stability and storage of plasma samples from patients treated with NOACs. Thus, laboratories may choose to perform their own studies and validate sample stability. However, assays such as the aPTT should be performed within 4 hours if the sample is maintained at room temperature. Therefore, as a conservative approach we propose that each coagulation testing on NOACs plasma samples should be performed within 4 hours if maintained at room temperature. For long-term storage, samples should remain at −20°C for no more than 2 weeks. If longer storage periods are required, samples can be maintained at −70°C or colder.

### 4.4. Assessment of NOACs in Plasma

Different routine coagulation tests could be used to estimate the intensity of anticoagulation in patients treated with NOACs. More specific assays are used to accurately estimate plasma drug concentrations using specific calibrators and controls. All of these tests present advantages and drawbacks that should be discussed in order to implement, in a clinical setting, the rational of using one or more coagulation tests to help clinicians in their daily practise.  Table 2 summarizes the coagulation assays that could be used to estimate either the plasmatic concentration of NOACs or the relative intensity of anticoagulation in specific situations.

#### 4.4.1. Global Coagulation Tests

*(1) Dabigatran: Activated Partial Thromboplastin Time (aPTT)*. The recent recommendations of the* Subcommittee of Control of Anticoagulation of the Scientific and Standardisation Committee* of the* International Society on Thrombosis and Haemostasis *(ISTH) mention that aPTT using most available reagents can be used to determine the relative intensity of anticoagulation due to dabigatran. However, this Subcommittee states that aPTT should not be used to quantify the plasma drug concentration and that further studies are required to determine the relative sensitivity of aPTT reagents to dabigatran. They add that each laboratory should be aware of the sensitivity of their aPTT assays to dabigatran and this can be achieved using commercially available plasma calibrators [31]. However, it is unknown if these specific dabigatran calibrators, used out of their specific platform context, are reliable calibrators that could reflect accurately the impact of dabigatran in plasma from patient's sample. Effectively, the aPTT is affected by numerous preanalytical and biological variables that could restrict the use of these calibrators since we ignore how these artificially spiked plasmas are prepared.

**Figure 1:**
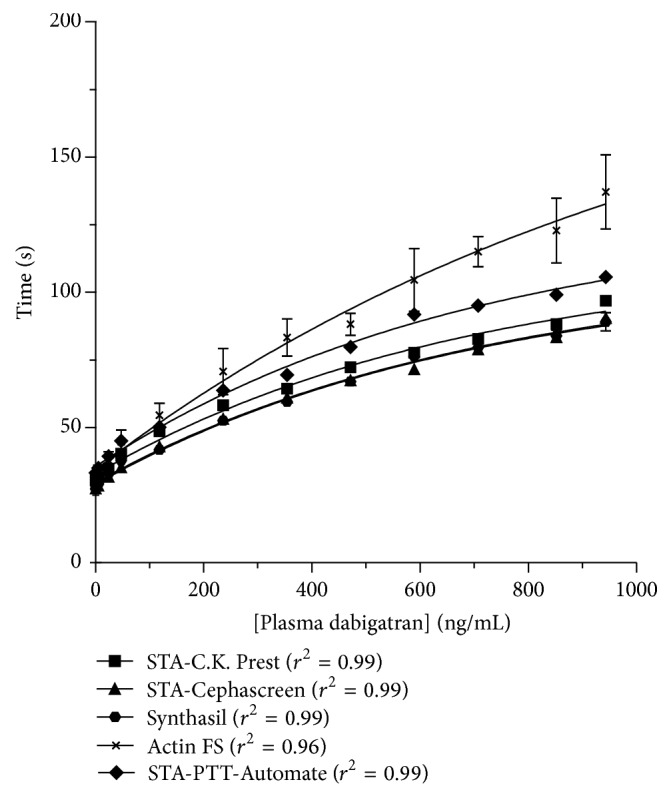
Impact of dabigatran on activated partial thromboplastin time (aPTT). There is a concentration-dependent prolongation of aPTT with a plateau at higher concentrations. The sensitivity depends on the reagent (*adapted from *Douxfils et al. [32]).

Thus, aPTT could provide guidance for the assessment of bleeding risk. For example, it is stated in the EU-Summary of Product Characteristics (EU-SmPC) that when dabigatran was used for the prevention of stroke in NVAF with a* bid* dosing regimen, an aPTT ratio greater than 2 × ULN (or an aPTT prolongation of about 80 seconds) at trough (10–16 h after the previous dose) reflected the 90th percentile of observations (i.e., 200 ng/mL at *C*_trough_) and is considered to be associated with a higher risk of bleeding [4]. While the EU-SmPC attempts to provide guidance on coagulation tests value associated with a bleeding risk, studies revealed that the interreagent variability prevents using an aPTT of about 80 seconds as reflecting plasma dabigatran concentration of 200 ng/mL [32] (Figure 1). For example, in one of previous* in vitro* studies, we found aPTT from 48.6 to 62.5 seconds for the concentration of 200 ng/mL, depending on the reagent. Similar observations have been demonstrated for the threshold proposed in prevention of venous thromboembolism regarding the bleeding risk [32]. Moreover, the reagent/instruments combination can also influence the sensitivity, increasing further the possibility to standardize the results among different laboratories [33]. Therefore, laboratories should be aware about the sensitivity of their aPTT reagents (respective to the instrument on which the test is performed) towards dabigatran. More recently, aPTT values from 50–90 seconds with dabigatran concentrations >200 ng/mL were found in patient's plasmas, and aPTT results could be as high as 75 seconds with only 50 ng/mL dabigatran in plasma [34–37].

Thus, aPTT is a global assay that does not accurately reflect plasma dabigatran concentrations, especially at higher values. It has limited sensitivity depending on the reagent and is not suitable for precise quantification of the anticoagulant effect for several reasons. First, the aPTT is affected by preanalytical (inappropriate collection, handling, and/or storage) and biological variables (lupus anticoagulant, hereditary or acquired factor deficiencies, hepatic insufficiency, vitamin K deficiency, disseminated intravascular coagulation, increased risk of thromboembolic events, hyperthyroidism, patients with diabetes, cancer, or myocardial infarction, and in pregnant women) [38, 39]. Secondly, a prolonged aPTT is not strongly predictive of haemorrhage and patients may experience bleeding while displaying a normal aPTT [39–41]. Finally, the dose-response is not linear, precluding the possibility to differentiate minor versus major overdoses (Figure 1). In definitive, we should rely on aPTT only when other more specific coagulation assays are not available. On the other hand, the main advantage of aPTT is that it is widely available and does not require specific laboratory experience. Importantly, this test could be the only one available to assess dabigatran therapy in nonexperienced hospital but each laboratory should know the sensitivity of its own reagent and deal with the several variables if aPTT is used in this context.

*(2) Rivaroxaban: Prothrombin Time (PT)*. The* Subcommittee of Control of Anticoagulation of the Scientific and Standardization Committee* of the ISTH mentions that PT, with a sensitive reagent, can be used to determine the relative intensity of anticoagulation in emergency situations when required but should not be used to quantify drug plasma concentrations [31]. Effectively, initial pharmacokinetic studies sponsored by the drug manufacturer revealed that PT could be a useful assay to assess the pharmacodynamics of rivaroxaban due to a close linear correlation between PT and plasma drug concentrations [42, 43]. However, the investigators of these PK studies already pointed out that rivaroxaban measurement should not be translated to INR values since INR was developed to normalize PT in patients treated by VKA using the International Sensitivity Index (ISI) [42, 43]. Recently, a proposal for a specific ISI_rivaroxaban_ has been investigated [44]. This method requires further investigations but could probably provide reliable standardization of the results obtained with the different thromboplastins [45].

**Figure 2:**
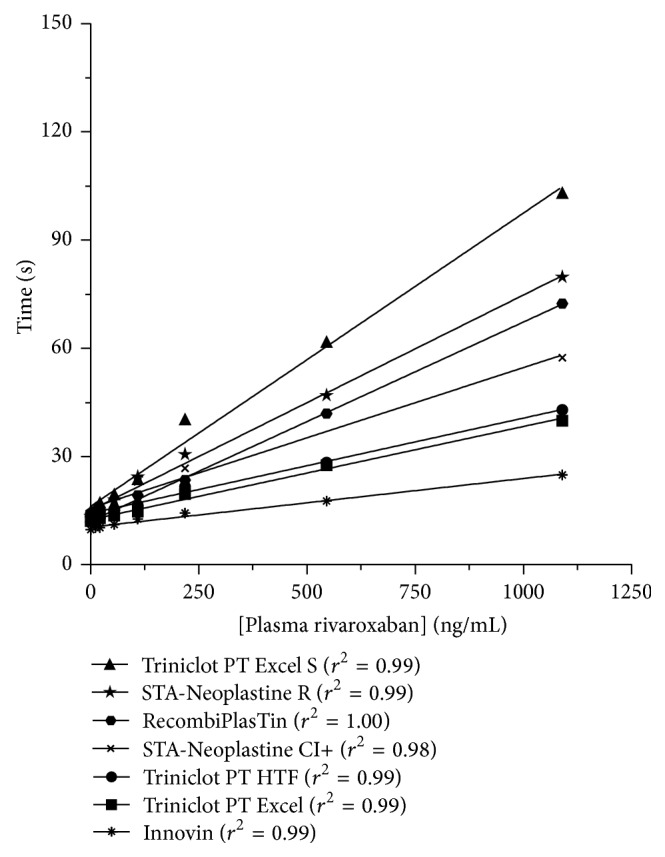
Impact of rivaroxaban on prothrombin time (PT). There is a linear concentration-dependent prolongation of PT. The sensitivity highly depends on the reagent (*adapted from* Douxfils et al. [49]).

In phase-II studies assessing the PK and PD of rivaroxaban, only two thromboplastins were assessed, that is, STA-Neoplastin and Innovin, a rabbit-brain and human recombinant thromboplastin, respectively [42]. These results already showed considerable interreagent variability in terms of sensitivity [42]. Therefore,* in vitro* studies aiming at investigating the between reagent variability have been proposed [46–49]. A large variability was reported suggesting that the laboratories should be aware about the sensitivity of their own reagent towards rivaroxaban (Figure 2). Importantly, as mentioned for dabigatran, the reagent/instrument combination can also influence the sensitivity and therefore it is recommended that each laboratory assess the sensitivity of their reagent on the instrument on which the test is performed [33]. The* Subcommittee of Control of Anticoagulation of the Scientific and Standardization Committee* of the ISTH supports this statement [31]. Nevertheless, one weakness of this approach is that commercially available calibrators are labelled to be used with its corresponding chromogenic anti-Xa assay. Therefore, as for dabigatran and the aPTT, the quality and the accuracy of these calibrators for the calibration of PT reagents are not warranted.

The use of a calibrated-PT was assessed in an* ex vivo* study that revealed a poor correlation between calibrated-PT and measured rivaroxaban plasma concentration [50]. Moreover, in the literature, one can find data showing that depending on the reagent, normal PT may be obtained with therapeutic concentrations of rivaroxaban [51] while some authors stated that it may be possible to exclude therapeutic level of rivaroxaban by using sensitive reagent (i.e., RecombiPlasTin 2G) [52]. We also observed that RecombiPlasTin 2G was more performing than other rivaroxaban-sensitive PT reagents (personal own data), showing that this reagent is probably less influenced by interindividual variability.

Therefore, depending on the reagent, PT must not be used to estimate rivaroxaban concentrations in plasma and reflects poorly the intensity of anticoagulation due to rivaroxaban. The poor sensitivity (especially for reagents like Innovin), the important variability (less pronounced with RecombiPlasTin 2G), and the poor linear correlation with the LC-MS/MS preclude the use of PT to estimate rivaroxaban concentration in plasma. In addition, drugs or hematologic abnormalities affecting at least one factor assessed by PT could bias the conclusions. However, as for the aPTT with dabigatran, PT is the most widely available test to assess the intensity of anticoagulation of rivaroxaban. It is available 24/7 and does not require specific laboratory experience. Importantly, results should be given as ratio or as seconds and should never be expressed as INR. In addition, the knowledge of the sensitivity of the reagent is mandatory. 

**Figure 3:**
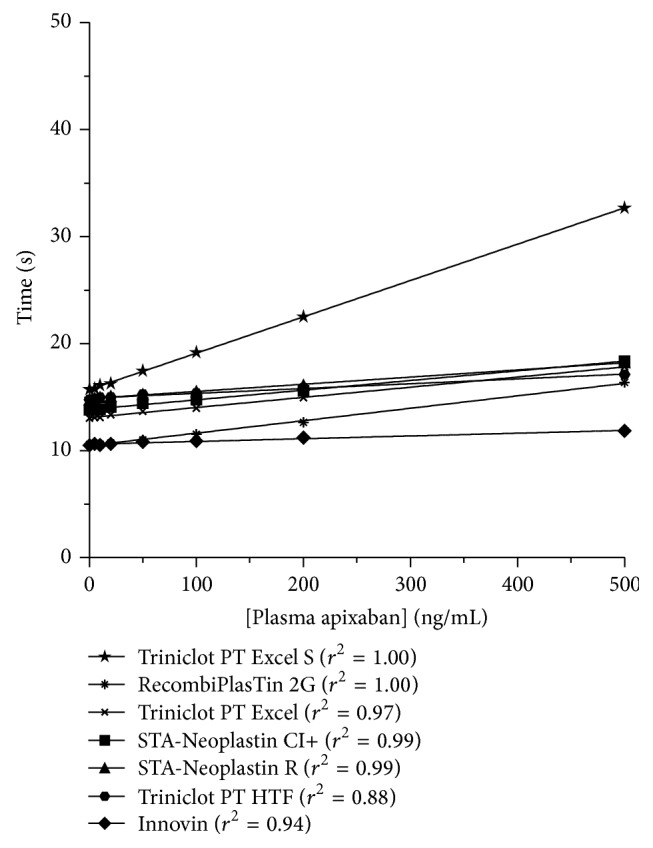
Impact of apixaban on prothrombin time (PT). There is a linear concentration-dependent prolongation of PT. The sensitivity highly depends on the reagent. PT is not sensitive enough to estimate plasma drug concentrations of apixaban or to assess the relative intensity of anticoagulation at therapeutic doses (±100 ng/mL at *C*_max⁡_ in healthy subject taking apixaban 5 mg bid) [56] (*adapted from *Douxfils et al. [55]).

*(3) Apixaban: Prothrombin Time (PT)*. The* Subcommittee of Control of Anticoagulation of the Scientific and Standardization Committee* of the ISTH elected to perform a collaborative study to analyse the anticoagulant effects of apixaban on several coagulation tests. The method includes the measurement of PT using RecombiPlasTin 2G and Technoclot reagents, the prothrombinase-induced clotting time (PiCT), and 5 chromogenic anti-Xa assays. They mention that all methods adequately determine the concentration of apixaban if added to normal human pool plasma* in vitro*. They also revealed that using assays procedures validated by manufacturers for individual coagulation platforms could further reduce error variances [53]. However, initial PK/PD studies in patients treated with apixaban assessed the effect of apixaban on coagulation with PT/INR, aPTT, and chromogenic anti-Xa assays [54]. INR values were prolonged modestly, on average increasing by 20–40% with RecombiPlasTin as reagent. aPTT increased minimally and did not differ significantly between the different apixaban dosing groups in secondary prevention of acute coronary syndrome (2.5 mg* bid*; 10 mg* od*; 10 mg* bid* and 20 mg* od*) [54]. As stated for rivaroxaban, INR must not be used for the assessment of apixaban while PT, expressed either in seconds or as ratio, is inappropriate to ensure an accurate quantitative measurement of apixaban [55]. Prothrombin time is not sensitive enough to ensure a quantitative measurement of apixaban at the plasma level obtained in initial PK/PD studies with the doses given in approved indications [54–56]. Moreover, depending on the reagent, PT may be normal with therapeutic concentration of the drug (±100 ng/mL at *C*_max⁡_ and 30 ng/mL at *C*_trough_ in healthy subject) [56, 57]. For the most sensitive reagents it may only inform the clinician if the patient is taking the drug. This interreagent variability (Figure 3) prevents valid recommendations of cut-offs in seconds associated with a bleeding risk applicable to all reagents [55]. In addition, drugs or haematologic abnormalities affecting at least one factor assessed by PT could bias the conclusions. We definitively do not recommend PT to estimate plasma concentration of apixaban.

*(4) Dilute Russell's Viper Venom Time (DRVV-T): A Useful Assay for All NOACs*. Russell's viper venom contains a potent activator of factor X and is usually used for the assessment of antiphospholipid syndrome (APS) [58]. It was demonstrated previously that rivaroxaban influences APS screening tests leading to false positive results, especially on the DRVV-T [59, 60]. A study suggested that DRVV-T could be used for the evaluation of all NOACs but this needs to be validated in patient samples [61]. Recent findings confirmed this hypothesis in plasma samples from patients treated with rivaroxaban [62]. It was demonstrated that the confirmed reagent, which contains the higher amount of phospholipids, should be preferred since it is not sensitive to the cases of APS. Moreover, it is more accurate than the screening reagent despite a small loss of sensitivity. However, validation with drug level measurements in plasma using LC-MS/MS and direct comparison of the effects of dabigatran and apixaban is lacking and needs to be investigated.

#### 4.4.2. Specific Coagulation Tests

*(1) Dabigatran: Diluted Thrombin Time (dTT), Ecarin Clotting Time (ECT) and Ecarin Chromogenic Assay (ECA)*. Assays used to evaluate thrombin functions are more sensitive than those that are not specific toward this coagulation factor [32, 63]. Thrombin time (TT) was demonstrated to be too sensitive for dabigatran [32, 63]. However, this may guide the clinician in the perioperative setting since a normal TT indicates no clinically relevant anticoagulant effect of dabigatran.

The strong sensitivity of TT towards dabigatran led to the development of a calibrated diluted thrombin time (dTT) using dabigatran standards to calculate the dabigatran plasma concentration. Thus, the Hemoclot thrombin inhibitor (HTI) was developed and has been proposed as a rapid, standardised, and calibrated assay to determine plasma concentrations of dabigatran [32, 35, 63, 64]. Theoretically, the use of diluted plasma (1/8 initially) allows the HTI assay to measure dabigatran effects at a wide range of concentrations. It is also normalized with normal pooled plasma, thus reducing the intraindividuals variation due to abnormalities in patient's plasma characteristics, especially the fibrinogen rate. In addition, there is a linear relationship between dabigatran concentrations and the dTT, which is suitable for the precise quantitative assessment of dabigatran [32, 35, 63, 64]. It is also fully automatable and has been adapted to different coagulometers in order to be easily implemented in laboratories. Several studies showed that the HTI highly correlates with dabigatran plasma concentrations measured by LC-MS/MS in patient's plasma [34–36, 63]. However, this test is limited by the fact that, in case of switching therapy (i.e., from heparins/heparinoids to dabigatran etexilate; or from hirudin and derivatives to dabigatran etexilate), it will be slightly influenced by the presence of such inhibitors in the plasma. This implies the necessity of an accurate anamnesis of the drugs taken by the patient to avoid overestimation of drug concentrations in plasma.

Nevertheless, for the accurate determination of dabigatran plasma concentrations below 50 ng/mL, the more sensitive LC-MS/MS method is still required [34, 35].

The ECT assay provides a direct measure of the activity of direct thrombin inhibitors. When dabigatran was used for the prevention of stroke in NVAF with twice daily dosing, an ECT of greater than 3 × ULN (or an ECT prolongation of >103 seconds) at trough reflected the 90th percentile of observations and was considered to be associated with a higher risk of bleeding [4]. While development of commercial kits might improve the practicality of this test, these kits have not been standardised or validated with dabigatran [63]. For these reasons, ECT cannot be recommended for emergency monitoring of anticoagulant effects. Moreover, ECT is not widely available and is known to have interlot variability indicating that calibration is also required with this test [32].

Recently, the ECA, the chromogenic variant of the ECT, calibrated with standard calibrators, has been proposed for the accurate measurement of dabigatran in patient's plasma with a lower limit of quantitation similar to HTI [34, 36, 65]. The ECT and the ECA both use the ecarin, a highly purified metalloprotease isolated from the venom of* Echis carinatus*, to activate prothrombin into meizothrombin [66, 67]. Meizothrombin can then cleave the fibrinogen to form fibrin (ECT) or can cleave a chromogenic substrate that release* paranitroaniline* (ECA). They present the advantage to be insensitive to heparins, as the heparin-antithrombin complex cannot inhibit meizothrombin due to steric hindrance. Therefore, only direct thrombin inhibitors could interfere with the test in case of switching therapy. Moreover, the artificial addition of prothrombin and the use of a chromogenic substrate in the ECA allow this test to be insensitive to abnormalities in patient's plasma [68]. Currently, the ECA and the HTI showed similar performances in different studies [34, 36, 65]. Head-to-head evaluations of these tests in the “real life” context, using the LC-MS/MS as reference measurement, are still required to compare the accuracy and reproducibility of these specific methodologies developed to measure dabigatran plasma concentrations.

*(2) Rivaroxaban: Chromogenic Anti-Xa Assays*. The initial study assessing the safety, PK, and PD of rivaroxaban suggested that rivaroxaban plasma concentrations and inhibition of FXa activity correlated closely [69]. Thus, investigations have been undertaken to optimise current chromogenic anti-Xa assays primarily used for the monitoring of LMWH [47–49, 70–75]. Thanks to specific calibrators and controls containing a defined amount of rivaroxaban, a dedicated chromogenic anti-Xa assay has been proven to accurately estimate the plasma rivaroxaban concentrations >30 ng/mL [50].

Another advantage of chromogenic anti-Xa assays is that they are less influenced than PT by the sample collection conditions and the variations in the amounts of clotting factors in patients. Several chromogenic anti-Xa assays are available on the market; however, only some of them are labelled to ensure the quantitation of rivaroxaban plasma concentrations. It is therefore important to work on specific coagulation platforms to reduce the CV in the interlaboratory setting [73]. The results from a multicentre trial involving 24 laboratories indicate that chromogenic anti-Xa methods, using rivaroxaban calibrators and controls, are suitable for the measurement of a wide range (20–660 ng/mL) of rivaroxaban plasma concentration [50, 76]. They also present a good repeatability and reproducibility and the estimated concentrations have been comparable with the concentrations measured by LC-MS/MS in a previous study [50, 75]. A limitation of chromogenic anti-Xa assays is that the standardisation across reagents and methods is not easily achieved but interesting results showed that interassay variability could be easily reduced [73]. Importantly, chromogenic anti-Xa assays, specific calibrators and controls, are not yet widely available, and their use may be difficult in emergency situations. However, the availability of liquid-stable reagents could improve the practicability of these tests.

Nevertheless, taking into account the lower sensitivity of chromogenic assays compared to LC-MS/MS and the variability of coagulation analysers that may further increase the imprecision at the lowest concentrations, detection and quantitation of lower levels (<30 ng/mL) in rivaroxaban treated patients still require LC-MS/MS analyses [50, 77].

The LC-MS/MS method is more accurate and is useful in the entire range of rivaroxaban concentrations. Consequently, the LC-MS/MS is required for quantification of very low to moderate rivaroxaban concentrations (3 to 30 ng/mL) in clinical samples.

*(3) Apixaban: Chromogenic Anti-Xa Assays*. Due to their good sensitivity towards the inhibition of FXa by apixaban, chromogenic anti-Xa assays calibrated with specific apixaban calibrators should be performed to estimate plasma drug concentrations [55, 57]. Another advantage of chromogenic anti-Xa assays is that they are less influenced than PT by the sample collection conditions and the variations in the amounts of clotting factors in patients [46]. Patients of the APPRAISE-1 study had participated in a PK/PD study suggesting that apixaban-mediated anticoagulant effect can be detected, even at very low plasma concentrations, using a standard laboratory chromogenic anti-Xa assay with either LMWH or apixaban calibrators [78]. However, what the authors failed to discuss in their paper regarding their data is that when comparing plasma apixaban concentrations estimated by the calibrated STA-Rotachrom and the true plasma concentration measured by LC-MS/MS, the chromogenic anti-Xa assay tended to underestimate the plasma drug concentration [78]. The French GEHT study, that assesses the reliability of different chromogenic anti-Xa assays for measurements of apixaban using specific calibrators and controls, revealed that the 3 chromogenic anti-Xa assays tested using lyophilised apixaban calibrators and controls correctly quantify a wide range of apixaban concentration. However, a limitation of this study was that it was performed in spiked samples and not from patients treated with apixaban [57]. Thus, further studies are required with validated calibrators that compare dedicated calibrated chromogenic anti-Xa assays with LC-MS/MS. As for rivaroxaban, it seems to be preferable to work on specific coagulation platforms in order to reduce the interlaboratory CV [57]. Nevertheless, confirmation of the accuracy of the quantitative measurement of apixaban by chromogenic anti-Xa assays using specific calibrators and controls should be confronted to the reference LC-MS/MS measurement in “real life” patients treated by Eliquis. In addition, chromogenic anti-Xa assays are not widely available, and their use may be difficult in emergency situations. However, as for rivaroxaban, the availability of liquid-stable reagents could improve the practicability of these tests.

## 5. Measurements in Other Media

NOACs can also be measured in other media such as urine or serum samples. Regarding the measurement in urine samples, it is important to mention that about 80% of dabigatran is excreted into urine as active compound and about 60% of rivaroxaban is cleared from circulation by glomerular filtration, 30% of which is excreted as active drug and the other part is metabolized in an inactive form before the renal excretion. To obtain results within minutes and possibly to develop point-of-care (POC) techniques, some authors have proposed measurement of dabigatran and rivaroxaban in urine samples [79, 80]. This testing may be useful for special patient populations such as those with acute deterioration of renal function due to any disease, before surgical interventions, during unexpected bleeding or thrombotic episodes while on therapy with NOACs, the oldest and youngest populations, pregnancy, suspicion of overdose, and intoxication. The test is based on the development of different colours in presence and absence of oral direct FXa and thrombin inhibitors. The strength of the methods is that they are not invasive and can be performed repetitively, that the results are available within 15 min, that the patients can perform the test themselves, and that the tests do not require standards and controls. The methods are described as sensitive, specific, accurate and possess a very high interrater agreement. However, the methods are not interesting in patients with creatinine clearance below 10 mL/min, confounders may also alter the identification of the colour generated, and the tests do not provide information on compliance. Importantly, these tests are qualitative and not quantitative and therefore cannot guide the physician on bleeding or thrombotic risks [79, 80].

The measurement of rivaroxaban and apixaban in serum samples has also been proposed [81]. The determination of rivaroxaban and apixaban from serum samples of patients may be beneficial in specific clinical situations when additional blood sampling for plasma and thus the determination of FXa activity are not feasible or results are not plausible. These tests are performed with chromogenic anti-Xa assays calibrated with homemade serum samples spiked with rivaroxaban or apixaban. Results revealed that the determination of rivaroxaban and apixaban from serum samples of patients can be performed with all chromogenic assays but some differences of the results between them exist. An adaptation of the chromogenic assays to current coagulation platforms seems feasible and may even improve the results but still remains to be tested [81].

## 6. Perspectives

The present review summarizes the information on how to handle clinical samples from patients treated with dabigatran etexilate, rivaroxaban, or apixaban in order to accurately measure plasma concentrations/effects of these compounds. Clearly, dedicated tests should be preferred when assessing these samples in order to avoid misinterpretation that could have clinical implications (e.g., with aPTT or PT the laboratory could refer the absence of NOACs in the perioperative setting while they are still present at clinically relevant concentrations). However, these specific assays are not widely available, are frequently time-consuming, and require the use of calibrators and controls that further increase the cost of these tests. In addition, while they are interesting for the measurement of normal to high levels of inhibitors, they seem unreliable for the measurement of low plasma concentrations that could be encountered in the perioperative setting. While the majority of the elective procedure can be performed with low residual plasma concentrations (<30 ng/mL for dabigatran and rivaroxaban [82]), high-risk surgeries may require a more accurate measurement of residual plasma level of these lowest concentrations. Therefore, there is a need to develop specific coagulation tests accurate in these low concentrations.

In addition, the development of a global coagulation test, which is sensitive to all NOACs and for which a cut-off associated with bleeding or thrombotic risk can be provided, is still needed. Finally, to date, the clinical benefit of such monitoring has not been proven yet. A large, sufficiently powered, clinical trial comparing standard treatments with dose-adjusted regimens in certain categories of patients is highly requested.

## 7. Conclusion

Up to now, no biological assay can be recommended for the assessment of all NOACs. Specific coagulation assays, depending on the NOACs, should be used in order to provide the more reliable information on plasma concentrations. The use of calibrated chromogenic anti-Xa assays and a dilute thrombin time should be recommended for the assessment of direct FXa inhibitors and direct thrombin inhibitors, respectively. Global coagulation tests, such PT and aPTT, are not useful at all and can lead to misinterpretation that could have clinical implications if the result is not fully understood.

The use of dedicated assays, using validated platforms, may probably improve the benefit-risk profile of NOACs by identifying poor- or high-responders. Monitoring such therapies that were claimed to be independent of any biological testing may be useful to provide guidance in case of bleeding, thrombosis recurrence, before urgent surgery or procedure, for populations excluded from clinical trials, and for those with several comorbidities. However and importantly, the clinical benefit of such monitoring still needs to be proven in a large, sufficiently powered, clinical trial designed to compare standard treatments with dose-adjusted regimen of these NOACs.

## Figures and Tables

**Table 1:** Summary of patients/situations that could benefit from point measurement of plasma concentrations.

(i) Bleeding or recurrence of thrombosis	
(ii) Before invasive procedure or surgery	
(iii) In patient with potential drug-drug interactions	
(iv) In patients with genetic mutations (e.g., rs2244613 minor allele carriers for dabigatran etexilate—no mutations are currently known for the other NOACs)	
(v) In patients with extreme body weight (<50 kg or >110 kg)	
(vi) In elderly patients (>75 years of age)	
(vii) In a case of accumulating interfering factors	

**Table 2:** Advantages and drawbacks of coagulation tests that could be used to estimate plasma concentrations of NOACs or to estimate the relative intensity of anticoagulation.

	Molecule(s)	Utility	Laboratory experience	Availability	Sensitivity^†^/ specificity	Dependence of the reagent	Cut-off for a risk of bleeding (unit(s) of expression)
aPTT	Dabigatran	Limited: poorly reflects the intensity of anticoagulation	Not required	24/7—all laboratories	±100 ng/mL/no	Yes	Yes: depends on the indication and the reagent (ratio and seconds)

TT	Dabigatran	Limited: only to exclude the presence of dabigatran. Useful in the perioperative setting	Not required	24/7—all laboratories	Too sensitive^‡^/no	Yes	Not established

dTT	Dabigatran	Proven: accurately estimates the plasma concentrations—results expressed in ng/mL	Required: requirement of calibrators and controls	Requirement of trained personnel—only in specialized laboratories	±10 ng/mL/no	No	Yes: depends on the indication (ng/mL)

ECT	Dabigatran	Limited: standardisation and validation required	Required: interlot variability probably requiring calibrators and controls	Requirement of trained personnel—only in specialized laboratories	±15 ng/mL/no	Probably not but an interlot variability has been reported	Yes: depends on the indication (ratio and seconds)

ECA	Dabigatran	Proven: accurately estimates the plasma concentrations—results expressed in ng/mL	Required: requirement of calibrators and controls	Requirement of trained personnel—only in specialized laboratories	±10 ng/mL/no	No	Yes: depends on the indication (ng/mL)

PT	Rivaroxaban	Limited: poorly reflects the intensity of anticoagulation	Not required	24/7—all laboratories	from ±100 to >500 ng/mL (depending on the reagent)/no	Yes	Not established

Chromogenic anti-Xa assays	Rivaroxaban/ Apixaban	Proven: accurately estimates the plasma concentrations—results expressed in ng/mL	Required: requirement of calibrators and controls	Requirement of trained personnel—only in specialized laboratories	±10 ng/mL/yes-no (depending on the anti-Xa assay)	No	Not established

DRVV-T	Dabigatran/ Rivaroxaban/ Apixaban	Partially proven: confirmation should be done in plasma samples from patients treated with dabigatran and apixaban	Not required	Only in specialized laboratories	±100 to 200 ng/mL (depending on the reagent and the molecule)/no	Yes, but less importantly than for PT or aPTT	Not established

aPTT: activated partial thromboplastin time; DRVV-T: dilute Russell's viper venom time; dTT: dilute thrombin time; ECA: ecarin chromogenic assay; ECT: ecarin clotting time; PT: prothrombin time; TT: thrombin time.

^†^Sensitivity is defined as the concentration required to double or to halve the clotting time (for chronometric assays) or the OD/min (for chromogenic assays), respectively.

^‡^Plasma concentrations of dabigatran >30 ng/mL frequently excess the maximal time measured on most coagulometers.
